# Editorial Department

**Published:** 1856-12

**Authors:** 


### EDITORIAL DEPARTMENT.

---

### *Transactions of the American Medical Association, for* 1856.

The ninth volume of the Transactions of the American Medical Association, being the proceedings of the session held in Detroit in May last, has just been received from the publishers, Messrs. Collins, of Philadelphia. We have been looking forward to the day of publication of this volume with no little interest, for we frankly confess we have a great affection for this organization, and shall experience the most poignant regret whenever we shall discover from its proceedings any abatement of interest in its behalf upon the part of the profession of our country; and it affords us great pleasure to state, in turning over the pages of this ponderous volume of unexceptionable typography, that it will suffer from no comparison with any previous volume, whether we regard the variety or range of subjects, or the manner in which they are treated.

This volume contains nine hundred pages, being some one hundred and fifty more than the previous volume, the numerous reports and scientific papers being extended through a hundred and thirty-five pages of this excess. We do not wish to be understood as intimating by this comparison of size, any intention of gaging the *quality* of the contents by the *quantity;* but in the profession there has always been a class of croakers who have prognosticated an early declension of interest in the welfare of the association among medical men, and consequently, a speedy termination of its existence. And to these may be joined those who may be found in our ranks, who have always affected to ignore its very existence, and strut, with amazing complacency, wrapt up in their self conceit, plainly intimating that there can be nothing good abroad in the land except it be the work of their hands, or is especially illuminated by the light of their countenances. We derive a heartfelt satisfaction when we are thus enabled to point so triumphantly to the evidence that the world may be made to revolve without the aid of their shoulders at the wheels.

As an evidence of the national character of the association, and that it may well claim to be the legitimate mouthpiece of the profession of the entire Union, we need only to point to the character and extent of the representation at the session at Detroit. At this meeting there were delegates from twenty states and one territory. There were represented eighteen state societies; eighty-two district, county and city societies; seventeen medical colleges, and fifteen hospitals. The entire number present being three hundred.

After so much by way of introduction, we propose to dip into the contents of the volume, and notice their character so far as our limits will allow.

We have already given a full report of the daily proceedings of the session, and shall not now stop to recapitulate the minutes of each day's transactions. We may, perhaps, be excused for referring to the Treasurer's Report, which proves that the association is somewhat of a "money institution." The receipts during the current year having been $3,584 26, leaving a balance in the treasury at the opening of the session, of $950 52.

The address of the president, Dr. George B. Wood, follows next in order, and for the first time, we have had an opportunity of reading it. It is a well written article, but being without any "text" as a starting point, or suggestor of thought, it partakes of the usual discursive character of such productions. It is eulogistic of the association, recapitulates the causes which led to its organization, recounts what it has accomplished, and exhorts it to a continuance of good works. The point in the discourse which will most particularly arrest the attention of the reader, is the repudiation of the term *allopathists* as applied to regular practitioners of medicine. We quote portions of this section:

"We have been called *allopathists*, in contradistinction to a sect of irregular practitioners who have taken to themselves the title of *homœopathists:* the latter term signifying that its professors treat disease by influences similar in their effects to the disease itself; the former that *other*, and of course dissimilar influences are used. It must be remembered, that the designation was not adopted by ourselves, but conferred upon us by Hahnneman and his followers.

"But we are *not* allopathists. The regular practice of medicine is based on no such dogma, and no exclusive dogma whatever. We profess to be intelligent men, who seek knowledge, in reference to the cure of disease, wherever we can find it, and, in our search, are bound by no other limits than those of truth and honor. We should not hesitate to receive it from the homœopathists, had they any to offer. We would pick it up from the filthiest common sewer of quackery; for like the diamond, it has this excellent quality, that no surrounding filth defiles it, and it comes out pure and sparkling, even from the kennel. This is the light in which the medical profession should present itself to the community. We are men who have sought in every possible way to qualify ourselves for the care of their health. We present them, in our diplomas, the evidence that we have gained sufficient knowledge to be trusted with this great charge; and we stand pledged before them to extend our knowledge and increase our skill, as far as may lie in our power. Membership in our honorable profession is the proof we offer that we are no false pretenders, no interested deceivers; but upright men, intent on the performance of our professional duties. This the people can understand. But when we designate ourselves as *allopathists*, they may well ask, in what are you better than any other medical sect, than the *homœopathists*, the *hydropathists*, the *Thomsonians*, the *eclectics?* Let us discard, therefore, the false epithet. Let us not only never employ it ourselves, but show that when applied to us by others, it is inappropriate and offensive, and that the use of it in future would be contrary to gentlemanly courtesy, and the proprieties of cultivated society. I say again, we are not *allopathists;* we are simply *regular practitioners of medicine*, claiming to be honest and honorable—in other words, to be gentlemen."

The address concludes by a reference to the increasing frequency of small pox, and urges the necessity of greater attention to the subject of vaccination, a matter of great public interest, and which is also discussed, at greater length, in one of the reports contained in this volume.

The scientific contributions to the volume, the legitimate fruit by which to judge of the healthful influence of the association, consist of seventeen reports and papers, beside the Prize Essay.

At the head of this column stands, as it most assuredly deserves to stand, the *Second Part of the Report on Deformities after Fractures, by Prof.* F. H. Hamilton, of this city. This paper resumes the subject where left in the Transactions of last year, and includes the consideration of fractures of the scapula, arm, forearm, and hand. One more paper, at least, will be required to complete the subject, with that minuteness of detail which is essential to the perfection of the design of the reporter. The elaborate character of the work upon which Prof. Hamilton is engaged, may be inferred when we state that this portion of his report alone, occupies one hundred and sixty-three pages of the volume. And we shall beg permission to turn aside here, from the regular routine of our remarks, long enough to gratify our pride as a Buffalonian, to call attention to the fact, that more than one quarter of the entire volume is occupied by two papers from the pens of a couple of the members of the profession resident in this city. And we think we may, with a good degree of sectional pride, point to what has been performed in behalf of the association, and the advancement of medical science, by our medical men. The volume of last year also contained two papers from their pens, and we would not forget that quite in the early days of the association,

Prof. Austin Flint was the author of the Prize Essay, and he was followed by Prof. J. C. Dalton, who subsequently became a professor in our Medical College, and for a time a resident among us, as the successful winner of the prize.

But we must return to Dr. Hamilton's report. We confess to no little difficulty in presenting a review of it, as that word is ordinarily accepted. The work is not one of startling theories, or the propounder of innovations in practice, which one may attack for the sake of argument; or praise, or blame, as the critic may seem inclined. It deals in facts, not fancies. Your judgment is not sought to be led captive by the subtilties of argument, but you have presented a cold, rigid array of stubborn facts, from which the deductions are self-evident, and you scarcely need the aid of the analytical tables, which are scattered through the report, to lead your mind to the conviction of the truth. With a careful, pains-taking labor scarcely conceivable, the minute history of every possible form of fracture, received under every variety of circumstance, and subjected to every mode of treatment, is here given, — with what is most important to the surgeon, the result of the treatment. This mass of facts is collected from every source that the industry of the reporter could make available. The grand problem which the doctor is working out, is the measure of success attending the treatment of fractures. And the benefit to accrue from the results of such labors, can be readily appreciated, after one has had the inestimable privilege of defending himself against a suit for malpractice, if his intellect is too obtuse to realize otherwise the benefits of all this toil.

Certain are we, that the pages of these reports will be thumbed by every unlucky doctor who may be the victim of a prosecution, with all the attendant horrors of an impoverished pocket and a ruined reputation staring him in the face, and for any gleam of comfort he may evoke from their contents will be ready to call down a blessing upon the author. We were informed last spring, that the evidence deduced from the first part of this report had been sufficient to preserve a physician, in one of our western states, from the consequences of a vexatious lawsuit.

Labors of so utilitarian a character, confer a positive and permanent benefit upon our profession, and entitle the performer to a position in the ranks of medicine and surgery far in advance of those whose only boast and claim is the performance of some dashing operation which derives all its eclat from its boldness, and requires naught but nerve and hardihood for its execution, and can boast only of a mangled corse for its trophy.

We should not do full justice to the report, did we omit to notice, that there is interwoven with the cases a *resumé* of surgical treatment, applicable to the different forms of fractures, so that when the work is completed, it will furnish a system of practice for the guide of the practitioner.

*Report on Hydrophobia.* By THOMAS W. BLATCHFORD, M. D., of Troy, N. Y., is the second paper in the series. There will be no one, we think, who examines its pages, to deny it the award of merit. It is the result of much labor expended in the collection of cases, one hundred and six in number, and all the facts to be derived from the analysis of the same, are distinctly brought out. The cases given have all occurred on this continent.

The committee say, "The main object of our inquiry has been to ascertain, if possible, whether hydrophobia prevails more at one season of the year than at another; whether the community is more in danger from the bite of rabid animals in hot weather and during 'dog days,' than during the colder months of the year; the proof, therefore, of the existence of rabies canina in any place, and at any time, is what we seek; and whether the virus is communicated to man or brute, the fact is equally apropos to the task assigned us, though not of equal importance to the community where it exists. We have confined our inquiries to the United States, because we thought the facts elicited would make a deeper impression upon the public mind the nearer they come to our own firesides, and because, as we shall see, by and by, the investigation has already been undertaken in Europe, and the results given to the public.

"The principal causes to which the origin of rabies has been ascribed, or which have been supposed to favor the development of the disease in *dogs*, are want of food, or extreme hunger; want of water, or violent thirst; putrid food; drinking stagnant water; climate; particular seasons of the year; intemperature of the weather, as the extreme rigor of winter, or the immoderate heat of summer; and in combination with the latter, *especially* a certain mysterious influence which Sirius, the dog star, is supposed to exert over the canine race. It is scarcely credible that such different and opposing causes should produce the same results without the coöperation of some other circumstances. The prevalent opinion seems to be, that the fiery dog star, who is in the ascendant in the northern hemisphere during the heat of summer, not only predisposes *dogs*, more than other animals, to become rabid, but that he also gives intensity to the heat of the atmosphere, which, with the putrid, or putrescent food and drink, or rather want of drink, excites a fever in his system, and infuriates him to madness. Although this sideral fancy is not endorsed by any medical authority, several authors assert and maintain the thermal hypothesis, and both appear specious and plausible, from the fact of their concurrence with most of the other alledged causes of rabies."

On the other hand, it has been asserted that rabies prevails most during the coldest weather of the winter season. A mass of evidence is contained in the report in favor of this view, as well as of the fact of the greater frequency of the disease in the northern latitudes. Rabies seems to be a rare disease in tropical climates. The disease is never seen in Egypt and Turkey, which are overrun with dogs; it "is not known in the Island of Cyprus, or Syria, bordering on the sea." It is extremely rare at the Cape of Good Hope, and in the interior of Caffraria. There was not a single case in Jamaica for a period of fifty years previous to 1783. It *was* equally rare in India; but now the disease is very common there. The apparent immunity from the disease in some tropical countries may, at first view, seem to be the effect of *climate*. That such is not the case, however, is probable from the fact that the disease has, in several instances, originated or been developed there, and when existing has spread as readily by inoculation as elsewhere. Such was the case in India, and also in the Island of Crete.

The report sums up the argument as follows:

"Although statistics of rabies go to show that, contrary to popular prejudice, it occurs most frequently in cold countries, and during autumn, winter and spring; still it appears that of the whole number of cases that occur out of the tropics, during the year, *nearly* an equal proportion occur during each month of the year, from which it may be inferred, that the appearance and prevalence of the disease, at particular seasons, and in certain localities or regions, are *accidental*, and in no way connected with, or produced by any thermal, or sidereal influence."

It has been satisfactorily proved by experiments, says the report, that putrid meat, or want of food or drink, never generates the disease in the dog.

"After this brief and hasty examination of the evidence for and against the generation or development of rabies, by the causes we have considered, we are driven to the humiliating acknowledgment, frankly avowed by the authors of the article on rabies in the French Dictionary of the Medical Sciences, 'that the true cause of the disease is not known, or very imperfectly understood.'"

From the fact that nearly every case of genuine rabies has been traced to the bite of some animal, the report concludes, "it is fair to infer, that *it never proceeds from any other cause*." The difficulty attending this assumption, is seen and admitted. It does not account for the *origin* of the disease, and compels us to fall back upon the strong probability, which has almost the force of positive proof, that the germ of the disease is in the animals themselves. Eight cases of hydrophobia are given, following the bite of animals not rabid, or not known to be so affected. One case (No. 92) which was fatal, the person was never bitten, but it was supposed the saliva was in some manner brought in contact with an abraded surface upon the hands. The depravation of the secretions by strong emotions, or passions, is referred to.

Very few cases of hydrophobia result from bites on parts of the body protected by clothing. The cases reported are almost uniformly from bites upon exposed parts of the body, such as the fingers, hands, face, nose, ears, &c., &c.

An interval of time elapses between the infliction of the bite, and the development of the disease: this is the period of incubation. The duration of this stage of incubation is made a subject of inquiry in the report, and the cases in reference to it are arranged in tabular form. The shortest period given, between the inoculation and development of rabies, is ten days; the longest, 365 days. The average period of incubation in 89 cases, was about 70 days, a much longer period than is usually supposed.

The average duration of the disease in 72 fatal cases in the human subject, was 3 days: 5 died on the 1st day; 20 on the 2d; 12 on the 3d; 27 on the 4th; 6 on the 5th; 3 on the 6th; 2 on the 7th; 2 on the 10th; and 1 on the 21st.

Nothing is said upon the subject of treatment, except what incidentally comes up when discussing the effects of the excision of the injured part. The opinions of several different authorities, are quoted as to its propriety, even after the interval of a number of days, and when the cicatrix has become swollen and painful.

Hydrophobia is a disease about as rare as fatal. During the year ending June 1st, 1850, twenty-six deaths are returned in the "Mortality Statistics of the Census of 1850." The seasons of the year were as follows: 1st quarter beginning with March, 7; 2d quarter, 7; 3d quarter, 3; and the last quarter, December, January and February, 9.

The report concludes by the recitation of cases. These are given in the language of the several reporters, and altogether, form the most graphic description of the disease possible to give, as well as an epitome of all the forms of treatment employed to relieve the sufferers from their horrible fate.

"*Report on the Causes which Impede the Progress of American Medical Literature.*" By S. D. Gross, M. D., and the "*Report of the Committee on Medical Literature.*" By R. J. Breckinridge, M. D., follow next in order. These two reports, or rather the debates which grew out of their presentation, were the most animated and spicy of the session. The reputation of their several authors, was sufficient of itself to command the undivided attention of the members, but it was understood they were to present antagonistic views upon the best means of fostering American Medical Literature; and both were eloquent advocates of their several opinions.

Dr. Gross opens his paper with a reference to the difficulties which environ his task, and to the risk he ran of exciting displeasure, or of having his motives and feelings misapprehended and misrepresented. Duty, however, compelled him to speak the whole truth. We make the following quotations from this portion:

"My motto shall be, 'My country first, my profession next.'"

"Have we a national medical literature? If so, what are its nature and extent?

"It requires no labored argument to answer these questions. Undoubtedly we have a national medical literature; but that its character and extent are not what they should be, or what we hope they ultimately will be, is equally true. It is an immature, an infantile literature, destitute of bone, and muscle, and sinew; gradually, but steadily developing itself, and destined, ere long, to take its place by the side of that of other nations. The medical literature of America was conceived in adversity, rocked in the cradle of sorrow, and reared on a diet of bread and water; and yet, as will appear by and by, it is not destitute of value to the possessor, or without honor to the giver. If it lacks the stately proportions of the medical literature of some of the more refined and cultivated nations of Europe, it possesses the vigor of a healthy and steady growth, surely, though slowly advancing to the full maturity of a sound and sturdy manhood."

After such an introduction, paragraphs of which we only have given, he traces the progress of American medicine, during the difficulties, dangers, and poverty of our colonial existence and the struggles of the revolution, causes sufficient in themselves to repress all effort to improve, or elevate the profession of the country. Up to this period, and, indeed, until shortly after the commencement of the present century, hardly any work deserving the name, had appeared on medicine from the pen of a native physician.

But a brighter day dawned. The early part of the present century supplied us with the writings of Rush and Barton, of Wistar, Dorsay, Chapman,

Coxe, Thatcher, and several other productions of minor note. As yet, the profession had not produced one solitary great work on any subject. Then appeared in pretty rapid succession, the valuable treatises of T. R. Beck, Gibson, Dewees, Horner, Eberle, Hare, Silliman; and at a still later period, those of Dunglison, Wood and Bache, N. R. Smith, Meigs, Wood, Dickson, Oliver, Paine, Condie, Bell, Warren, Stewart, Ray, Gerhard, Bartlett, McLean, Pancoast, Morton, Miller, Mitchell, Frost, Henry H. Smith, and others. The last few years have been unusually prolific in valuable monographs.

He thinks no one can accuse the profession of the United States, of to-day, of idleness, ignorance, or indifference, as the issues of the press, the existence of forty medical periodicals, and nearly forty medical schools, would amply attest their industry and zeal.

Having brought such evidence to prove what the profession has accomplished, he classifies the causes which still obstruct the progress of our national medical literature as follows:

"1st. The identity of the language of this country with that of Great Britain. 2d. A disposition in the profession to patronize English works in preference to American. 3d. A want of independence in our periodical press. 4th. A lack of industry in observing and recording facts in private and hospital practice."

We have not time, or space, to give in detail the arguments adduced in support of these positions. He refers to the prevalent use of English works, principally republications, on this side of the water; the prominence given to them as text-books in our colleges; the injury inflicted thereby upon native authorship in withholding from them a suitable return for their labors both in honors and pecuniary rewards. That while British publications find such willing publishers and ready sale in this country, the only American works of any note that have ever been republished in Great Britain, are Dorsey's *Surgery*, Beck's *Medical Jurisprudence*, Ray's *Treatise on Insanity*, and Warren's *Observations on Tumors.*

He cites numerous American productions and gives the most flattering testimony of their ability to abide a comparison with the foreign productions, and insists that they are entitled to a preference by the American physician and student. But we can pursue the analysis no farther.

Dr. Breckinridge's paper opens with a catalogue of the most important additions to American medical literature for the year. Having disposed of the list, he adds:

"In a critical survey of our national medical literature, not only for the current year, but for years gone by, we have been strongly impressed by two antagonistic particulars respecting it: 1st. Its general excellence; and 2d. The depreciation which it has so often met with at the hands of some of our brethren. It will be found that scarcely a solitary reporter to your body on this subject, has failed to lament the low state and condition of our professional literature, and to suggest explanations more or less satisfactory, of such condition, and means, more or less profound, of remedying it."

He pursues the subject in a strain of eloquent diction not frequently met with in the discussions of a subject of so grave and prosy a character as that of medicine. He points to the successes of native authorship in tones sufficiently exultant to gratify the intensest American pride.

Having left scarce anything to be desired by the American author at the hands of his brethren, he proceeds to discuss the propriety of an international copyright law, and the restrictions to be imposed thereby upon the circulation of the works of foreign authors.

A calm consideration of the question, in its most obvious and important aspects, leads him to distrust the propriety and wisdom of any such law; and it is far from clear that, even in giving all their weight to the *private* interests involved in this question and no higher interest be considered, the authors of books are entitled to demand exclusive property in them from their own government, much less the right to claim such a monopoly from foreign states.

Growing bold in his arguments for the universality of knowledge, and that it should have free course, he proceeds to raise doubts whether copyright, or exclusive property for a limited period, is really politic and wise on the part of governments when dealing with their own citizens. It is, however employed, a tax upon knowledge, an obstruction to the general progress of literature and science. The pursuit of literature is not a trade, but a glorious calling,—glorious, we presume, although it will not purchase a patch for the tattered garments of its poor slave.

So far as they go to show what has been accomplished in the field of our national literature, these reports do not differ, but, one maintains we have toiled, but without reward: the other, that we have received a just, if not full recompense.

Our Americanism prompts us to sympathise with our struggling countrymen, although it be in the toils of literary bondage; and we much prefer to see them enjoy some more substantial fruit of their labors, than the *plaudits of posterity!* We can hardly believe that the arguments of Dr. B., will meet with a response in the hearts of the majority of his readers. It is the eloquence of the rostrum, and displays the orator, but contact with the stern realities of every day life, soon arouses other emotions than simply those of pleasure, evoked by the magic of rhetoric. The life we live is of to day, and however sweet may be the assurance of an immortality of fame, hunger pinches, and the cold wind chills with as keen sensations as if there existed no future. Present reward will palsy no man's hand, and corroding want will fire no soul with enthusiasm. Starvation may goad him on, but it will be with the incentive only of a goad, and not of the hope of an immortality. The plaudits of generations yet to come will fall powerless on dead ears, and awaken no emotions in a pulseless heart.

*Report on Plans of Organization for State and County Societies.* By A. B. Palmer, M. D.

This is an earnest appeal in favor of the formation of local medical societies, and furnishes numerous suggestions as to the manner in which they may be best made subservient to the advancement of science. Accompanying the report are forms of constitutions for state and county societies, which doubtless will be found useful, and if used lead to a uniformity in the manner of organization of the several societies.

*Report on the Changes in the Composition and Properties of the Milk of the Human Female, Produced by Menstruation and Pregnancy.* By N. S. Davis, M. D.

The title very fully describes the design and the character of the paper, and is the result of observations and analyses made by Dr. Davis himself. He has collected and examined by the microscope, and by chemical analysis, specimens of milk from healthy females while nursing, without the secretion being disturbed by menstruation or pregnancy; and again when lactation has been accompanied by menstruation; and again in cases where lactation has been complicated by pregnancy. Figures are given illustrating the microscopical appearances of these several conditions of the milk, and tables exhibit the results of the chemical analyses.

The summing up of these investigations we give in Dr. Davis's own language:

"If we may deduce conclusions from the limited number of observations and analyses here detailed, we may find a very definite answer to the question under consideration, so far as it relates to the changes in the *composition* of the milk.

"1st. The occurrence of pregnancy during lactation, produces a very marked diminution of all the solid or nutritive constituents of the milk, such diminution continuing to increase as the pregnancy advances.

"2d. In examining the separate proximate constituents, it will be observed that a much greater relative diminution takes place in the casein, the butter or oil, and the salts, than in the sugar and extractive matter.

"3d. There appears to be added to the milk secreted during the progress of utero-gestation, some of the *granular* bodies or colostrum corpuscles, and numerous minute infusoria or animalcular germs, which have been very rarely found in healthy milk.

"4th. Changes, the same in kind, take place in the milk secreted after the establishment of regular menstruation, but much less in degree; and the relative diminution of the several constituents is more uniform."

That these changes in the qualities of the milk affect unfavorably the health of the nursing child, he illustrates by the recitation of cases.

*Report on the Sanitary Police of Cities.* By JAMES M. NEWMAN, M. D.

Dr. Newman's report occupies some fifty pages, and that to good purpose. The author is favorably known as the writer of a Sanitary Report of the City of Buffalo, for the cholera year of 1854, and as a frequent contributor to our pages. As a general remark upon this production, we may say that it evinces much labor in condensation, a kind of labor which makes little impression upon the careless reader, but which is fully appreciated by any one familiar with the toil incident to the reduction of statistics to an available form.

At the outset, Dr. Newman declines to go over the old ground of sewerage, paving and ventilation. That, he says, has been already done at the instance of the association; and he pays an honorable tribute to his predecessors in the work. The object of his report is in his own language "to tabulate the effects of disease, and to exhibit by figures the ravages that *preventable* disease is committing in our midst, and especially in our cities." He affirms that epidemics have consumed too much attention, and that in the healthiest season a large proportion of deaths occur unnecessarily, but still unheeded.

Fixing upon two per cent., or one in fifty, as the normal average of deaths in circumstances favorable to life, he proceeds to show that the proportion in cities is far above this. The laborious calculations by which these results have been reached (in many instances by tedious deductions from census statistics) are evidences of the patient research and thoroughness of detail which characterize the author. In London, he finds a mortality of 1 in 39; in New York, of late years, about 1 in 33; in Philadelphia, 1 in 38 to 1 in 48; Baltimore, 1 in 36 to 1 in 50; Charleston, 1 in 25 to 1 in 52; Chicago, 1 in 21 to 1 in 43; New Orleans, 1 in 10 to 1 in 24. The variations, alone, in these tables, are sufficient to prove the preventability of much of this mortality; but the report also shows that for every death about 28 cases of sickness occur, and in like proportion is *this* preventable, and the suffering and expense entailed by it unnecessary.

Proceeding with his subject, Dr. Newman makes the distinction between zymotics and other forms of disease the boundary line of capacity for prevention. Of the 323.023 deaths in the United States, for the year ending June 1, 1850, about 41 per cent. were from zymotics. This general result does not stand alone. By reductions of each of the states, and of many returns from cities, this ratio is confirmed. He then enters into the question of the ultimate preventability of zymôtic disease, and discusses it in a broad, philosophical way, which does credit to him, not only as a man of figures, but one competent to their translation. One by one the principal zymotics are taken up, and their mode of propagation considered, calling to his aid a large mass of information not accessible to the general reader, and which we are glad to find condensed in an accessible form. Dr. Newman cannot be said to positively assert that all zymotic disease is abstractly preventable, though we think his reasoning, or at any rate his figures, all go to prove that startling and important position. Certainly, small-pox has been proven to be in this category, and typhoid and typhus fevers are almost equally so. However, Dr. Newman rarely makes an extravagant assertion, and he probably preferred to trust to the natural deductions of his readers.

Coming to means of prevention, the reporter starts with the proposition that personal appeals, education of the masses, and individual labor, are all insufficient to the sanitary reform of cities; that the poor, chained down to their hard lot by circumstances beyond their control, are not in possession of the means of reform; and that the task must be confided to a liberal and judicious sanitary police. In this, it seems to us, he strikes at the root of the matter, and arguing that if a city has the power to control the erection of buildings in relation to risks by fire, it has the same power in relation to risks by fever, Dr. Newman meets the question in the form to which it must finally come, after the failure of all temporizing measures.

We can only enumerate the special agents of health which he insists upon as matters of municipal care and responsibility.

Water leads off. He discards wells as a source of supply for cities; denies the policy of entrusting the water-works of a city to a private company; and urges that it should be introduced in the very quarters of the town where it *will not* pay, because there it will do most good.

Pure air comes next. After a forcible description of the evils incident to the tenant-houses of cities, he finds a remedy in the proposition, "that the evils are to be corrected, and corrected alone, by the strong arm of the law." The cupidity or the ignorance of landlords will forever prevent any reform coming from them; they build to rent, not to make healthy and happy; and we who live in cities need not be told that tyranny of capital, which extorts brick without straw, demanding labor where it gives in return neither light nor air, nor opportunity for cleanliness, is one of the great wrongs of our social life.

Dr. Newman discusses also to some extent, the upheaval of the soil in the summer season, as a matter for the interference of sanitary police. In this connection he quotes and endorses the well known arguments of Dr. E. H. Barton, of New Orleans, and the description of the Ellicott street epidemic, in this city, so forcibly given by Prof. Hamilton, four years since.

The subject of small-pox closes the body of the report. To it, and its prevention, Dr. Newman has given much attention, and is himself the author of some valuable statistics on the powers of vaccination.

In an Appendix, he gives in full the report of the committee of the New York Legislature on Tenement Houses in New York and Brooklyn. It is a forcible argument in favor of the ground taken as to the necessity of legal interference.

The report of Dr. Newman is on e of the most interesting in the volume and involves many questions of high practical importance. Well-written, clear and forcible in the progression of the argument, it was recognized at the meeting in Detroit, as one of the strong features of the occasion, and we are glad to find that its perusal warrants the encomiums there pronounced.

*Report on the Treatment of Cholera Infantum.*

This paper is fortunately short. *Fortunately*, we say, for we must honestly confess we can discover in it nothing particularly to commend, although from the first few opening lines we are led to expect a valuable addition to our stock of knowledge of this disease. It is illustrated by not a single new pathological fact, or by the recitation of a case. Its etiology of the disease is vitiated air, or malaria; pathology, derangement of the biliary apparatus; treatment, calomel and ipecacuanha, and all the usual remedies of the books.

Common-place essays on diseases, although enlivened by a quotation or two from Hippocrates, or Celsus, add nothing to the reputation of the author by a publication in such company.

*On the Use and Effect of Applications of Nitrate of Silver to the Throat.* By HORACE GREEN, M. D.

This report opens with a short historical sketch with references, of the rise and progress of the practice of local medication in the diseases of the Throat and Air Passages. The author then proceeds, under distinct heads, to consider the Effect of Nitrate of Silver in Follicular Pharyngo-laryngeal Disease, — In the Treatment of Membranous Croup, — Of Acute and Chronic Laryngitis, — Of Hooping-Cough, — Of Spasmodic Asthma, — Of Tuberculosis, following or complicated with Bronchial Inflammation.

Under this last head he refers to the success of the topical medication of thoracic diseases, during a period of eighteen months, effected by means of the operation of *catheterism* of the air-passages, or the injections of a solution of the nitrate of silver into the bronchial division.

He says, "During the last eighteen months, or since October, 1854, over one hundred patients, embracing cases of both pulmonary and bronchial disease, have been treated by this form of topical medication, conjoined with appropriate general remedies."

Of the results, he speaks as follows: "Of one hundred cases of thoracic disease treated by catheterism of the air-passages, seventy-one of the sum total are recorded as cases of *tuberculosis.* Of this number, *thirty-two* were considered cases of *advanced phthisis* — cases in which tubercular cavities were recognized in one or both lungs; and *thirty-nine* cases of *early phthisis*. Of the first division — advanced phthisis — *fourteen* have since died. *Twenty-five* were more or less improved; their lives being apparently prolonged by this method of medication. *Seven* only of the thirty-two cases of advanced phthisis were not benefitted by the injections.

"Of the *thirty-nine* cases of incipient tuberculosis, *twelve* of this disease have apparently recovered. *Five* more of this number are now, or were, at the time of making the report, in the enjoyment of a good degree of health.

"With respect to the above twelve cases, I say apparently cured; for, although the appearance of these patients, as manifested both by the physical and rational signs, is indicative of an ordinary degree of health, yet in a disease like that of tuberculosis, every medical man is aware that one year is a period too brief to speak decidedly with regard to the positive and final result."

Dr. Green and his nitrate of silver treatment, have been the subjects of a good many pretty sharp words during the last few years, but we think the critic will be captious indeed who finds much for fault-finding in this report. It is a calm, dispassionate summing up of what has been, and may be said in favor of the topical application of this remedy, and in this effort, at least, we cannot perceive that the report is justly chargeable with hobby-riding. Experience alone can test the absolute value of the remedy, and experience will ere long settle the question definitely. The doctor is evidently an enthusiast in this matter, and is, perhaps, not at all times as unprejudiced a witness as he believes himself, or desires to be. If the doctor could convince the profession that he handles the probang with all the dexterity and effect that he claims, he and his brethren would have but very few matters about which to differ.

*Report on the best mode of rendering the Patronage of the National Government Tributary to the Honor and Improvement of the Profession.* By Joshua B. Flint, M. D.

This paper discusses the impropriety and injustice of granting patents for quack medicines; suggests that Congress should place at the disposal of the American Medical Association sums of money, to be expended in the publication of their Transactions and costly works; for the distribution of prizes, &c.; recommends that the chiefs of the medical staffs of the army and navy be selected from the profession of the country, and not from the corps of army and navy surgeons; the appointment of a medical chief to the head of the marine hospital department; suggests alterations in the manner of selecting army and navy surgeons, with many other suggestions of a like character.

The various Reports on *Topography*, *Epidemics*, *Meteorology*, and *Mortality*, we are compelled to pass by.

The Reports of the Committees

*On Education.* By Wm. H. Anderson, M. D.,

*On Strychnia: its Physiological Properties, and Chemical Detection.* By Lewis H. Steiner, M. D.,

*Upon a Uniform System of Registration of Births, Marriages, and Deaths, and the Causes of Death.* By G. S. Palmer, M. D.,

Together with the Prize Essay,

*On the Arterial Circulation: its Physiology and Chief Pathological Relations.* By Henry Hartshorne, M. D.,

Close the series and the volume. But we have not time, or space, to speak of them at the length which their several merits demand. We must content ourselves with the taste we have given our readers, and refer them to the [illegible]

